# Co‐Design Workshops to Develop a Psychosocial Support Service Model for Refugees in Sweden Affected by Gender‐Based Violence

**DOI:** 10.1111/hex.14177

**Published:** 2024-08-12

**Authors:** Anna Pérez‐Aronsson, Elin Inge, Heba Alanbari, Iman Alsalamah, Miras Ghannoum, Zozan Abu Mohammad, Frida Johansson Metso, Frida Holmqvist, Johanna Belachew, Tove Filén, Frida Pålsson Hennoks, Anna Sarkadi, Georgina Warner

**Affiliations:** ^1^ Child Health and Parenting (CHAP), Department of Public Health and Caring Science Uppsala University Uppsala Sweden; ^2^ Centre for Women's Mental Health During the Reproductive Lifespan—WOMHER Uppsala University Uppsala Sweden; ^3^ Swedish Red Cross Competence Centre for Rehabilitation of Torture and War Trauma Stockholm Sweden; ^4^ Information Sweden County Administrative Boards of Västra Götaland Gothenburg Sweden; ^5^ Kvinnofridsmottagningen (Outpatient Clinic for Women Subjected to Violence), Uppsala University Hospital, Region Uppsala, and National Centre for Knowledge on Men's Violence Against Women (NCK) Uppsala University Uppsala Sweden

**Keywords:** co‐design, gender‐based violence, intervention development, mental health, patient and public involvement, psychosocial support, refugee

## Abstract

**Background:**

Experiencing gender‐based violence (GBV) is common among refugees. Intersecting systems of oppression can increase the risk of GBV and of suffering detrimental consequences, while concurrently creating barriers to meaningful support. Despite this, refugees with lived experience of GBV are rarely involved in the development, planning and adaptation of services and policies.

**Methods:**

This article reports on a formative research process that aimed to involve public contributors (refugee victim‐survivors of GBV) and relevant stakeholders in co‐designing a service model aimed at improving psychosocial support in Sweden. Led by a partnership of public contributors and academic researchers, the research process consisted of iterative cycles of co‐design workshops, complemented by scoping of existing literature.

**Results:**

The co‐design process resulted in a characterisation of the psychosocial service system needs, as perceived by the survivor co‐researchers and stakeholders, and a two‐level empowerment and support service model. The model included (i) a community‐based intervention to promote help‐seeking and (ii) psychosocial group support delivered in specialist clinics. Outcomes of the project included perceived benefits for those involved, service‐led direct changes and acquisition of funding for continued research on the co‐designed model.

**Conclusion:**

Improving psychosocial support for refugees in Sweden affected by GBV requires safe spaces to connect with peers and familiarise with available services, laws and rights in the society. Further, strengthened collaborations across sectors are necessary to meet the variety of needs. Co‐design workshops were an effective way to initiate changes in the service delivery model for psychosocial support for refugees in Sweden affected by GBV.

**Patient or Public Contributions:**

This is a participatory reflection on a participatory process. The survivor co‐researchers contributed to designing and carrying out the PPI process and have co‐authored this manuscript.

## 1 Introduction

Gender‐based violence (GBV) entails acts and threats of physical, sexual, mental or financial harm directed against someone based on socially ascribed gender differences [1]. It can occur within families and communities or be perpetrated by representatives of states and institutions, including different forms of violence such as intimate partner violence (IPV), non‐partner sexual violence and forced marriage [1]. It is a human rights violation [2] carrying devastating consequences for those affected and high costs for the society [3]. It is also recognised as a global health issue, estimated to affect at least one in four women worldwide [4] and associated with health problems such as chronic pain [5], perinatal death [6] and symptoms of post‐traumatic stress disorder (PTSD) [7] and depression [5, 7].

Although GBV occurs in all societal groups [4], it is rooted in gender norms and power inequities; consideration is needed of how intersecting systems of oppression shape experiences of GBV and create barriers to meaningful support [8]. Research reports that experiencing GBV is common among people with refugee experience (hereafter referred to as refugees) [9, 10] who often face similar social vulnerabilities as other groups disproportionately impacted by GBV such as ethnic minorities [11, 12] or groups with low socioeconomic status [9, 13]. Migration is understood as a social determinant of health, influencing and influenced by other determinants [14], with the health and well‐being of refugees impacted both by premigration trauma and by negative post‐migration social conditions such as poor employment opportunities, (relative) poverty, inadequate housing, social isolation and discrimination [15, 16]. Consistently, refugees have been described as experiencing GBV across the migration journey, including in resettlement, in different forms and by a variety of perpetrators such as armed forces, police, community leaders, asylum professionals, employers or family members [9, 17, 18]. This includes forced marriage, sexual violence and other forms of exploitation due to circumstances such as lack of protection, unsafe migration routes, financial difficulties and insecure housing [9, 17, 18]. Changes in power or family dynamics and gender roles, along with post‐migration stressors and mental health difficulties, can contribute to an increased incidence of IPV or an exacerbation of existing IPV [17, 19].

Two reviews on help‐seeking for IPV found that migrant and refugee women experienced similar barriers as those reported by women in general populations, but amplified by factors such as immigration laws, isolation and racism [12, 20]. Economic hardship and fear of deportation can create barriers to support [9], and experiencing racism can prevent IPV disclosure and help‐seeking [12, 20]. Healthcare encounters can be complicated by the need for an interpreter, which may be due to unavailability, the interpreter's lack of competence in working with IPV or the challenges of building relationships when communicating through a third person [20]. Other research has highlighted a lack of training among welfare personnel and moral discomfort in how refugees with experiences of violence are met by welfare services in Norway and Sweden [21]. Moreover, an exploration of broader service provision for refugees in Turkey and Sweden who have experienced GBV found variations in how services understand violence [22].

The barriers identified in a systematic review of refugees' access to mental healthcare and psychosocial support in European countries [23] have similarities with findings on help‐seeking for GBV. These include discomfort discussing certain topics with practitioners due to mistrust, a lack of appropriate language services and collaboration between healthcare services, a lack of awareness about services and treatment possibilities among refugees and a lack of awareness about physical symptoms that could indicate mental illness among practitioners. A Swedish study found that clinicians perceived the provision of trauma‐informed care to refugee women to be prevented by incorrect assessments, constraints in admission criteria, long waiting times, transportation difficulties, confidentiality concerns and multiple health conditions and healthcare contacts [24]. Further, lack of childcare, fear of having children taken into custody and complex psychosocial living situations complicated treatment.

The exclusion of refugees from the development, planning and adaptation of services and policies is understood to be part of the social conditions driving health disparities between refugees and the general population [25]. Research from Turkey and Sweden indicates that the health and social care needs of refugees with experiences of GBV do not currently inform the design or development of service provision [22]. Meaningful involvement of refugees as public contributors in health research—that is, research conducted ‘with’ or ‘by’ members of the public rather than ‘to’ or ‘about’—could improve policy and practice and have positive health impacts [26]. Bringing refugees and other stakeholders together in research examining different perspectives on problems and solutions could facilitate learning and action‐oriented outcomes [26]. Before embarking on service development research, researchers need to identify the most significant gaps that are feasible to tackle within the research context and timeframe. Involving public contributors in this process has the potential to diversify ideas regarding research priorities [27]. The range of perspectives on what should be investigated can streamline the allocation of time and resources, leading to more meaningful outcomes [27].

## 2 Methods

### 2.1 Study Aims and Design

**Figure 1:**
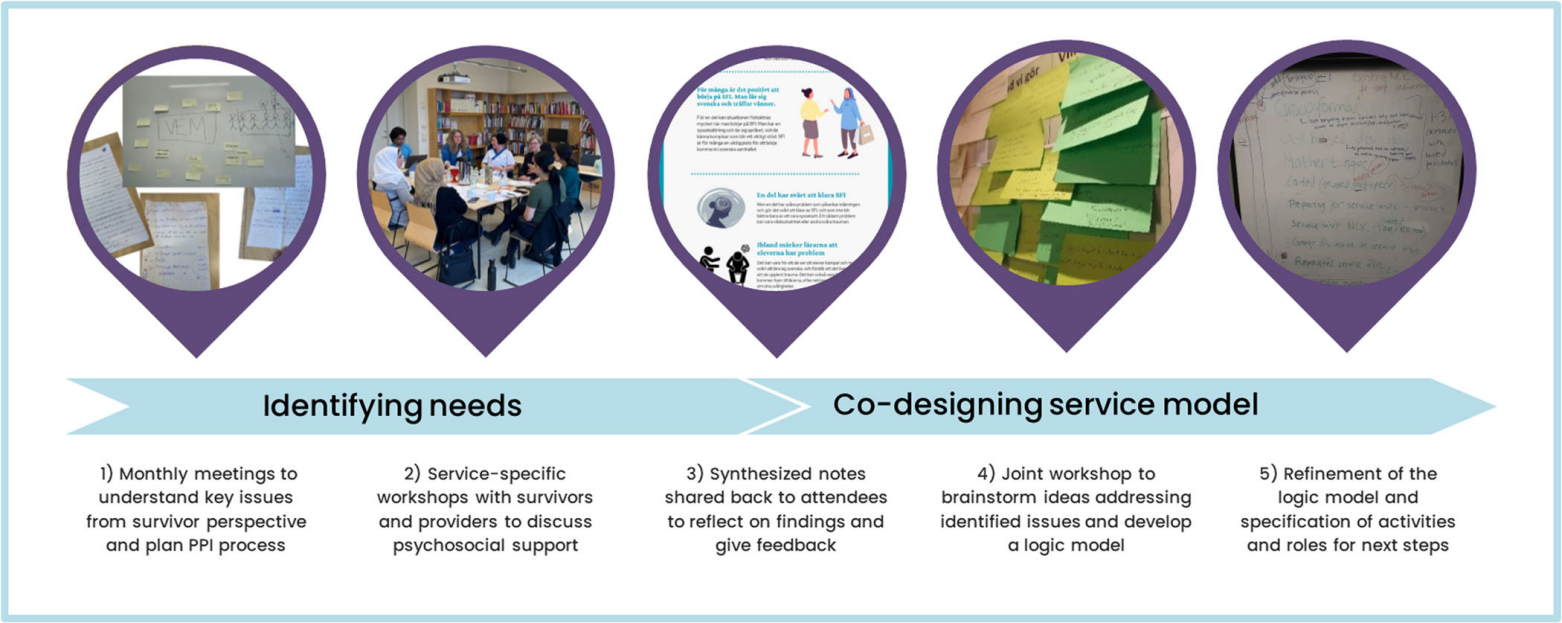
Overview of the PPI process.

This article reports on a formative research process that aimed to involve public contributors and relevant stakeholders in co‐designing a psychosocial support service model for refugees in Sweden who have experienced GBV. It was led by a partnership of academics and public contributors using an experience‐based co‐design approach [28]. The end goal was to construct logic models of various components to guide service re‐design and future evaluation. The documentation from the process, such as workshop notes, constitutes the empirical material that informed the service model development with existing literature informing the model refinement. Figure 1 gives an overview of the patient and public involvement (PPI) process, which consisted of iterative cycles of workshops with public contributors and service providers complemented by scoping of literature. The GRIPP2 checklist [29] guided our reporting of the PPI process. By publishing this process, we also aim to provide an example of involving public contributors in the early stages of the research cycle and of the various research and service initiatives that can come from collaborations between public contributors, service providers and researchers.

### 2.2 Setting—The Swedish Context

This study took place between August 2021 and October 2023 in mid‐Sweden, a high‐income country in northern Europe [30]. Historically regarded as a private matter, GBV has been reframed as a public concern in Sweden with increasing political prioritisation and responsibility placed on governmental organisations and services [31, 32]. Social services are tasked with supporting women exposed to violence, such as providing safe houses and counselling [33]. Healthcare is obliged to have routines for identifying violence and for taking appropriate action when violence is suspected [34]. Substantial work is done by NGOs, with the women's shelter movement having a long tradition of advocacy and support work [31]. Sweden's efforts to prevent GBV extend to the Migration Agency, with their webpage stating that migrants should tell staff if they have experienced violence to be offered support, information and referral to help [35]. People with a residence permit in Sweden are entitled to the same healthcare and social services as other residents [33]. Asylum‐seekers have the right to maternity care, contraceptive counselling, abortion care, a health examination and ‘care that cannot be deferred’ [36]. Those with a next‐of‐kin residence permit can, ‘in certain cases’, be granted an extended residence permit if their relationship ends due to IPV [37].

Despite being regarded as one of the most women‐friendly and gender‐equal societies in the world [38, 39], GBV continues to be a problem in Sweden with the prevalence of IPV reported to be similar or higher than in other European countries [40]. Scholars have discussed potential explanations, such as progress having benefitted different groups of women unequally and discrepancies between gender equality ideal and reality [39]. Research shows experiences of time constraints and regional differences in Swedish social services' provision of support to women with experiences of IPV [32]. Further, racist discourses have been argued to shape the conceptualisation of GBV in Swedish society, with non‐Whites, particularly Muslims, being depicted as ‘non‐gender equal’ and homophobic [38]. It has also been pointed out that, having traditionally positioned itself as a voice for antiracism, Sweden has statistically become highly segregated [38]. This pattern is particularly clear in the residential and labour markets and appears to be ‘inherited’, extending to Swedish‐born people with one parent from a non‐Western country. Moreover, the Swedish asylum law has been restricted in the last decade, starting with temporary restrictions introduced in 2016 [41] after a peak in asylum‐seekers mainly from Syria, Afghanistan and Iraq [42]. These restrictions made temporary residence permits the rule and limited family reunion possibilities [41]. Since then, restrictive asylum policies have continued to be implemented [43].

### 2.3 Forming a Steering Group With Public Contributors

We extended an invitation to participate as a public contributor in research on refugees' access to support for GBV through an Arabic‐speaking research assistant's networks at community sites such as Swedish for Immigrants (SFI) schools and associations for refugee women. We selected community recruitment over recruitment from a specific service as that might have limited understanding of user experiences and perspectives relevant to barriers to support. Four women (H.A., I.A., M.G. and Z.A.M.), self‐identifying as having relevant lived experiences, expressed interest. They had individual meetings with A.P.A., the PhD student who would facilitate the PPI process, to learn more about the research and the role of a public contributor. All four chose to become involved and will hereafter be referred to as ‘survivor co‐researchers’, a term they chose to reflect the shared ownership of this project and to emphasise their resilience and desire to help others survive as they have survived. From a researcher's perspective, we understand ‘co‐researcher’ to come with a commitment to value the diverse knowledge of all involved and use collaborative and ethical co‐design processes [44]. We will use ‘victim‐survivors’ or ‘refugee victim‐survivors’ when referring to the wider target group (i.e., refugees with experiences of GBV). Like Goicolea [45], we use victim‐survivors to acknowledge that people who have experienced GBV might feel both as victims and survivors. Using only ‘victims’ might be disempowering and paternalistic, whereas only ‘survivors’ might neglect certain experiences and contribute to creating a responsibility for those affected to overcome GBV. We held an initial group meeting using the domains of the Public Involvement Impact Assessment Framework (PiiAF) record card [46] to discuss the purpose of our research, our approaches to involvement and underpinning values and practicalities such as support needs. We agreed that the four survivor co‐researchers and the researchers G.W. and A.P.‐A. would form a team to run the project in partnership (hereafter referred to as the ‘steering group’). We recruited an interpreter together, who we agreed to engage as a collaborator throughout the PPI process.

### 2.4 Steering Group Positionality

The PhD student (A.P.A.), who facilitated the PPI process, is the daughter of a refugee from the Global South and an SFI teacher. She is a trained medical doctor who has been educated and worked in the Swedish healthcare system. During medical school, she volunteered as a guide for Spanish‐speaking undocumented migrants in healthcare encounters. The senior researcher (G.W.), who supervised the process, has a background in psychology and came to Sweden as a migrant from the Global North. The survivor co‐researchers (H.A., I.A., M.G. and Z.A.M.) are all women from Arabic‐speaking countries—mirroring one of the largest groups seeking asylum in Sweden in recent years—but their experiences of migration and GBV vary, as do their family situations and socioeconomic conditions. A second senior researcher (A.S.) was not involved in the steering group meetings but co‐supervised A.P.A. and participated in part of the PPI process. She is a professor in social medicine with clinical experience from asylum health clinics, who came to Sweden as a migrant from the Global North. We approached this research with a shared human rights–based understanding of everyone's equal right to health, participation and a life free of violence.

### 2.5 Co‐Design Process

#### 2.5.1 Step 1: Steering Group Discussions to Prioritise and Plan Research

During Step 1, we met monthly in the steering group to build relationships, identify key issues and priorities from a user perspective and plan the PPI process. We discussed the heterogeneity of experiences among refugee victim‐survivors and that GBV was a complex societal problem needing actions at various levels. We agreed to focus on developing suggestions that could reach broad groups of refugees and be implemented within existing nationally available services. Based on this, we identified seven services from which we recruited staff to participate in the PPI process. This included services tasked with providing societal information, offered nationally and regularly accessed by large groups of refugees as part of the Swedish reception system, and services with nationwide reach and competences important for providing psychosocial support. Table 1 gives an overview of the involved stakeholders. Demographic data were not collected as the work described in this article was PPI activity, meaning the survivor co‐researchers and providers were involved as partners and not research participants. However, the providers' professions were known because they were involved in their professional capacity. When referring to the full team involved in the process (i.e., survivor co‐researchers, researchers and the various service providers), we will use the term ‘co‐design team’.

**Table 1:** Overview of the stakeholders involved in different steps of the PPI process.

Stakeholders	Description	Participation	Members
Asylum Health Clinic	Offering a one‐time voluntary appointment for asylum‐seekers and newly arrived refugees, consisting of health examination and information about the Swedish healthcare. Nationally offered and regularly accessed by large groups of refugees as part of the Swedish reception system.	Steps 2–3	1 midwife 2 nurses
Civic orientation (CO)	A minimum of 100‐h‐long course with societal information for refugees and those arriving as next‐of‐kin to refugees. Organised by the municipalities and delivered in different languages. Nationally offered and regularly accessed by large groups of refugees as part of the Swedish reception system.	Steps 2–5	1 manager 3 course leaders (from different sites)
‘Information Sweden’—governmental webpage	Offers societal information in various languages through a governmentally funded webpage directed to migrants and asylum‐seekers, and develops material for civic orientation courses. The webpage address is spread by services such as the Migration Agency, public libraries, health clinics and open preschools.	Steps 2–5	2 staff members
Municipal open preschool	Nationally offered to all parents with children between 0 and 5 years. Free of charge and requires no formal enrolment, parents can choose when to visit. Aims to strengthen families by serving as a meeting place for children and parents and offering societal information and courses on topics such as child safety. This includes aiming to reach refugee mothers and their children and work to increase their participation in society.	Step 5	1 manager 1 educator
National Centre for Knowledge of Men's Violence Against Women (NCK)	A national centre commissioned by the government to increase knowledge of men's violence against women. Arranges training for professionals and runs a national helpline and a specialist clinic in Uppsala for women exposed to GBV. The phone number is spread through a variety of organisations including the Migration Agency.	Steps 2–5	1 gynaecologist 1 midwife 1 nurse 1 psychologist
Researchers	Researchers within public health, with background in medicine and psychology.	Steps 1–5	1 PhD student 1 associate professor 1 professor
Survivor co‐researchers	A group of women self‐identifying to have lived experience relevant to the research.	Steps 1–5	4 co‐researchers 1 interpreter
Swedish Red Cross (RC)	Competence centre and treatment clinics for rehabilitation of torture and war trauma, with nationwide reach. Visits at the clinics are free of charge and available to both asylum‐seekers, undocumented migrants and those with residence permits. Arranges training for professionals who meet refugees.	Steps 2–5	1 social worker 3 psychologists 1 physiotherapist (from different sites)
Swedish for Immigrants (SFI)	Swedish courses for immigrants with residence permits, organised by the municipalities. Nationally offered and regularly accessed by large groups of refugees as part of the Swedish reception system.	Steps 2–5	2 teachers (from different sites)

During the latter part of Step 1, our steering group prepared workshop material and rehearsed the format. A.P.A. drew on field notes from our PPI discussions and existing research [9, 10, 17, 19, 47] to create two ‘personas’, a design thinking tool, to be used in the workshops with providers to represent and stimulate understanding of user experiences [48]. The rest of the steering group and one of the co‐authors (A.S.) read the personas to ensure they captured important issues for refugee victim‐survivors in Sweden from experiential, clinical and academic perspectives. We developed a discussion guide for the workshops, which was guided by the Matching Needs and Service [49] and System Dynamics Mapping [50] methodological approaches focused on support needs, pathways to services and factors influencing access to support (Table 2). The guide aimed to help stakeholders express how they viewed their part of the wider system, support conceptual modelling by shifting the focus to the whole system and encourage stakeholders to identify connections and unintended impacts within the system [50].

**Table 2:** Discussion guide for the service‐specific workshops mapped against corresponding elements of the System Dynamics Mapping [51] and Matching Needs and Services [49] methodological approaches.

Discussion guide question	Corresponding component
Which support do you think [PERSONA] needs?	Matching Needs and Services
Do you think [PERSONA] will seek help? – If yes/no, why?	Alternative pathways Blocking factors Conversion factors
Would [PERSONA], or other refugees with similar problems, come in contact with [SERVICE]? – If yes/no, why? – What do you think will happen if [PERSONA] seeks support? – Where do you think they need to go to get support?	Alternative pathways Blocking factors Conversion factors
If [PERSONA] would come in contact with [SERVICE], what do you think would happen?	Matching Needs and Services Blocking and conversion factors Dwell time stages
Is there anything you would like to add?	Extra comments

#### 2.5.2 Steps 2 and 3: Workshops With Providers to Identify Needs

We held a series of service‐specific workshops bringing together survivor co‐researchers and providers to reach a shared understanding of improvement needs in current service provision. These workshops commenced with a round of presentations, clarifying the purpose of the workshops and group rules, and an opportunity to ask questions. This included reminding the attendees that the workshops were a public involvement activity—meaning everyone was there as collaborators in the project and not research participants—to collaboratively identify areas where access to support could be improved and subsequently co‐design a model aimed at improving service access. We recognised that the attendees were experts in different capacities—experiential, clinical and academic—and the diverse forms of knowledge were equally valued and important for the process. Then, everyone was given time to individually consider the personas and write down reflections before the group discussions. The survivor co‐researchers and providers reflected on factors that could influence refugee victim‐survivors' access to support, drawing on their different expertise, and the researchers contributed with insights from existing literature. Two people (E.I. and a medical student) took extensive notes during the group discussions. These were collected together with notes from the individual reflections, post‐its and photos.

Step 3 took place iteratively after each workshop and consisted of A.P.A. writing a brief summary of the collected material and sharing back with the attendees for feedback, through meetings and e‐mail correspondence. Through this, we gradually identified prioritised improvement areas to focus on and an initial vision of the model we would develop. When Steps 2 and 3 of the PPI process were completed, our steering group created a final summary of the identified needs and proposed a focus for the model. This was presented to the wider team at the joint Step 4 co‐design workshop.

#### 2.5.3 Steps 4 and 5: Co‐Designing of Service Model

Step 4 took place in a full‐day workshop gathering survivor co‐researchers, researchers and providers from the involved services. We commenced with presenting the results from previous steps, to consolidate findings and serve as a starting point for brainstorming ideas aimed at addressing the identified issues. The brainstorming took place in smaller groups, using a logic model template to facilitate systematic consideration of the key components of change, the relationships between them and the overall vision [52]. We had planned group formations beforehand, taking into consideration group dynamics and differing experiences and expertise. Each group wrote down ideas on colour‐coded post‐it notes corresponding to domains of the logic model. The groups presented their ideas to the whole team, gradually adding each group's contributions to a printed wall‐sized copy of the logic model template. E.I. and a medical student took notes of the discussion. The workshop included a celebratory aspect to recognise the work done so far and planning of the continued work including an invitation to co‐author the academic paper and discussion of dissemination strategies.

In Step 5, our steering group continued to develop the logic of the co‐designed model. A.P.A. and G.W. grouped post‐its with similar content together, forming key components. Then, the steering group discussed the model, refining it through critical reflection on the generated ideas, considering context, feasibility and potential risks. This included revisiting the notes from Steps 2 and 3 to crosscheck how the model addressed the identified challenges, and consultation of relevant literature [53, 54, 55, 56, 57, 58]. We identified a concern regarding reach and, consequently, recruited the municipal open preschools to be involved in the continued project and further refinement of the model. Step 5 also included establishing smaller groups to continue working on related research proposals.

### 2.6 Ethical Considerations

This co‐design process consisted of public involvement activities, meaning an ethical application review and informed consent process were not applicable. The survivor co‐researchers received an hourly reimbursement for their work as suggested by the National Institute for Health and Care Research [59]. The process was guided by literature on ethical co‐production [44, 59, 60, 61], and underpinned by consideration of the well‐being and safety of the survivor co‐researchers. Striving to be healing‐informed, key guiding principles included agency and empowerment; researcher preparedness and accountability; transparency and recognition; mutual exchange and relationship building [60, 61]. To exemplify how we put these principles into practice, we recruited several survivor co‐researchers to make it easier to opt out from activities if desired and include more people with lived experiences than academic researchers in the steering group to balance power. We conveyed that the survivor co‐researchers would not be required to disclose their experiences but contribute as experts of lived experience, and designed the process to enable this. Our initial discussions included prompting the survivor co‐researchers to consider how the process could be designed to enhance safety and reflect on support needs they might have as well as which actions to take if need of support would present. We took care to get to know each other within the steering group before the workshops with service providers and continued meeting in the smaller steering group throughout the process. Meeting times and venues, and forms of communication, were chosen together and we engaged the same interpreter throughout as we felt it was important for group dynamics. To promote continuous reflections and the possibility to modify our process, we conducted formative self‐evaluations using the PiiAF record card and A.P.A. kept a reflexive journal, recurrently discussing the process with the rest of the steering group and with E.I. (a co‐author doing a PhD on involving refugees as public contributors in research).

## 3 Results

**Figure 2:**
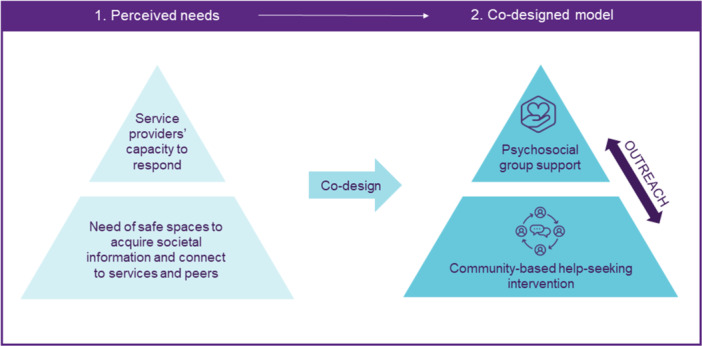
Overview of the outcomes of the formative research process.

The co‐design process resulted in a characterisation of the psychosocial service system needs, as perceived by the survivor co‐researchers and stakeholders, and a two‐level empowerment and support service model (Figure 2). The perceived needs and components of the service model are presented later.

### 3.1 Perceived Needs

Our co‐design team was in agreement that lack of awareness about available services, laws and rights in the society was a key issue. In particular, those arriving on next‐of‐kin visas or refugees on parental leave were known to take part in establishment services to a lesser extent [62]. This lack of awareness was perceived to contribute to a lack of trust in societal services, as it could facilitate misconceptions and rumours, whereas the support these services could offer might not be known. Fear of having your children taken into custody by social services was highlighted as a prominent concern. Our co‐design team raised that staff at services regularly accessed by refugees and where trust might be established, such as open preschools or SFI schools, would normally lack training in GBV and trauma. This could make it challenging to handle disclosures or distress. The clinicians in our team reported that there could be challenges in providing support to women coping with both experiences of GBV, conflict‐related trauma and precarious post‐migration social conditions. They requested strengthened collaborations with other sectors and more evidence of effective interventions. Our co‐design team also agreed that policy‐related barriers could influence access to support. Thus, the team agreed that while efforts to provide information, outreach and dialogue around available support were important and desired, improving the situation and access to support for refugee victim‐survivors also warranted actions to examine and address policy‐related harm. Furthermore, our co‐design team discussed that racialised minority victim‐survivors—including refugees and Swedish‐born individuals—might experience different forms of racism and discrimination in the Swedish society including in service encounters. This included, but was not limited to, Islamophobia, structural racism and interpersonal racism. Racism was perceived to shape narratives around GBV and culture and influence help‐seeking and access to support negatively. Thus, the team agreed that research and training efforts focused on the intersections of racism and discourses on GBV were needed.

We agreed that we needed to focus on particular aspects of the identified issues. We decided to focus on developing a two‐level empowerment and support model aimed at providing safe spaces for refugees to *access adequate information*, *connect with peers*, *familiarise with services* and increase *service providers' capacity to respond*. Not only did the stakeholders in our team express a strong need for such a model but we also deemed the development and subsequent testing and refinement of the model as feasible to pursue within our co‐design teams' competencies and collaborations.

### 3.2 The Co‐Designed Two‐Level Empowerment and Support Service Model

The two levels of our co‐designed model are as follows: (i) an intervention to promote help‐seeking delivered via regularly accessed community sites as part of existing civic orientation activities and supported by GBV training for service providers, and (ii) the delivery of a stabilising psychosocial group intervention to women with experiences of GBV and migration at specialist clinics. Figure 2 gives an overview of the model, which is described in more detail later.

#### 3.2.1 A Community‐Based Intervention to Promote Help‐Seeking

This aspect of the model was designed in response to the need for societal information and establishing trust. The intervention was developed to create safe spaces for refugees to acquire societal information according to their own agendas, from peers as well as through sensitively conducted service outreach. It was designed to be delivered as a group‐based module embedded into existing recurrent civic orientation activities at trusted community sites: as an extension of the lecture on violence in near relationships delivered at CO courses and as an addition to the civic orientation groups hosted by open preschools. Group‐based delivery was recommended as it could enable normalisation and peer support, and as questions asked by more outspoken participants could be beneficial for more reserved participants. Universal delivery at these regularly accessed community sites was regarded as helpful to avoid the stigma related to interventions directed only to certain individuals and to reach refugees with undisclosed experiences of GBV. Further, it could contribute to an increased awareness of GBV and related services, rights and laws among a wider group of refugees and thereby potentially facilitate future peer support and prevent violence. An additional benefit of building on existing civic orientation activities was that these already included information and service outreach necessary for improving social conditions, such as contacts with employment offices and housing services.

Our co‐design team perceived that brief information on a one‐time occasion was insufficient and that standardised information might not address circumstances and concerns specific to the refugee and asylum‐seeking context, such as fear of deportation. Therefore, the help‐seeking intervention was proposed to consist of recurrent and dialogue‐based sessions using specifically developed vignettes. The vignettes should depict a variety of GBV experiences within the migration context as well as in the general population to promote a nuanced understanding of GBV and avoid perpetuating cultural stereotypes. They would be used in the group sessions to gradually prompt discussions on the impacts of GBV, legislation and rights, available services and societal responses, the role of bystanders, personal safety and child protection. Our co‐design team endorsed vignettes as they would give people the possibility to ask questions about the person in the vignette rather than themselves.

A discussion format was favoured to allow participants' own agendas and priorities to shape information and to allow sharing of insights between participants rather than having information imposed by group leaders. Delivery in different languages was regarded as essential so participants could express themselves freely. The group leader's role would be to create a safe group environment, be respectful of differential views and experiences and ensure the discussions highlighted the intended topics. Co‐leading by peer facilitators was proposed as a beneficiary for trustworthiness and cultural responsiveness. Merely providing information about societal responses and services was perceived as insufficient to overcome distrust. Service outreach (i.e., meeting professionals such as psychologists or social workers at community sites) was regarded to have the potential to facilitate trust and decrease help‐seeking barriers. It was therefore included as a key component of the intervention, designed to take place after having allowed time to build positive relationships with the group facilitator and within the group.

To enhance safety and facilitate needed referrals, the module was to be supported by regular meetings between the group leaders and professionals from the specialised services participating in the outreach activities (i.e., psychologists and social workers), in which indications or disclosures of GBV would be discussed. To enable victim‐survivors to privately disclose violence and support needs, it would include a secure disclosure process, planned to be further developed as part of the refinement of the model.

**Figure 3:**
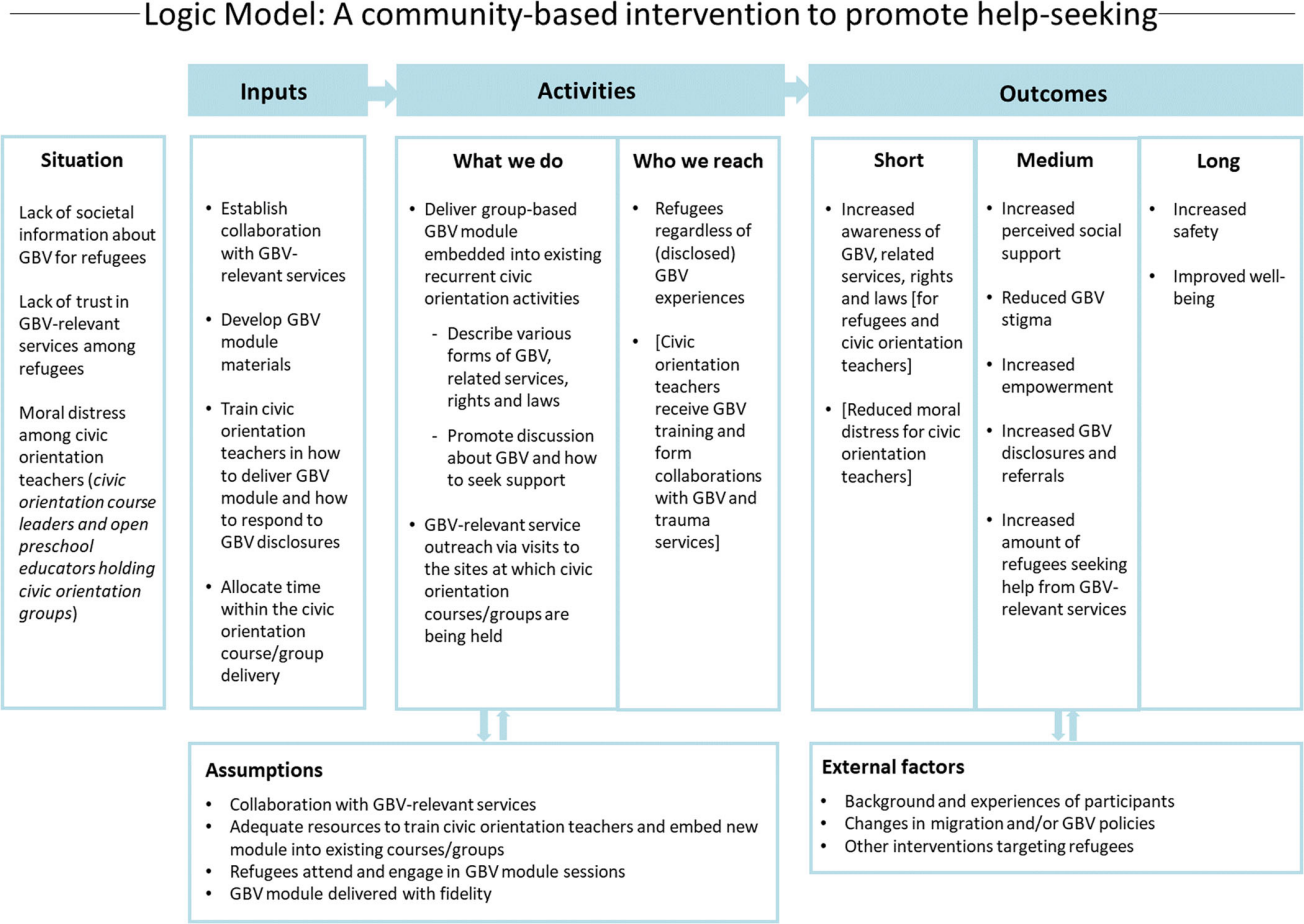
Logic model of a community‐based intervention to promote help‐seeking.

GBV and trauma training for municipal staff delivering the intervention was regarded as essential to develop and implement alongside the help‐seeking promoting intervention, to increase their capacity to facilitate the group discussions and respond to potential disclosures. The training was proposed to build on NCK's existing training materials with adaptations for the specific context. It should include different forms of violence and social vulnerability, the role of racism, non‐judgmental listening and knowledge of available societal support and possible referrals. This training component was not further developed in this PPI process as we identified that further investigation of attitudes and current training would be required first. See Figure 3 for a logic model of a community‐based intervention to promote help‐seeking.

#### 3.2.2 Psychosocial Group Support Delivered in Specialist Clinics

This aspect of the model was developed in response to the identified need for increased knowledge of psychosocial interventions for individuals who have experienced multiple traumas and face difficult social circumstances. Because the proposed community‐based intervention would aim to promote help‐seeking, it could, if effective, increase the demand on specialist services. Therefore, our co‐design team regarded efforts to increase specialist services' capacity to provide psychosocial support to refugee victim‐survivors as essential to develop alongside the help‐seeking intervention. The researchers and clinicians in our co‐design team conducted a review of literature on psychosocial group interventions for migrant women with experiences of GBV. The focus on group‐delivered interventions was chosen as our team regarded the potential to promote normalisation and social support as important to explore. The clinicians, researchers and survivor co‐researchers discussed the content and format of the identified interventions and agreed to test group‐delivered Skills Training in Affective and Interpersonal Regulation (STAIR) for women with experiences of migration and IPV [63].

**Figure 4:**
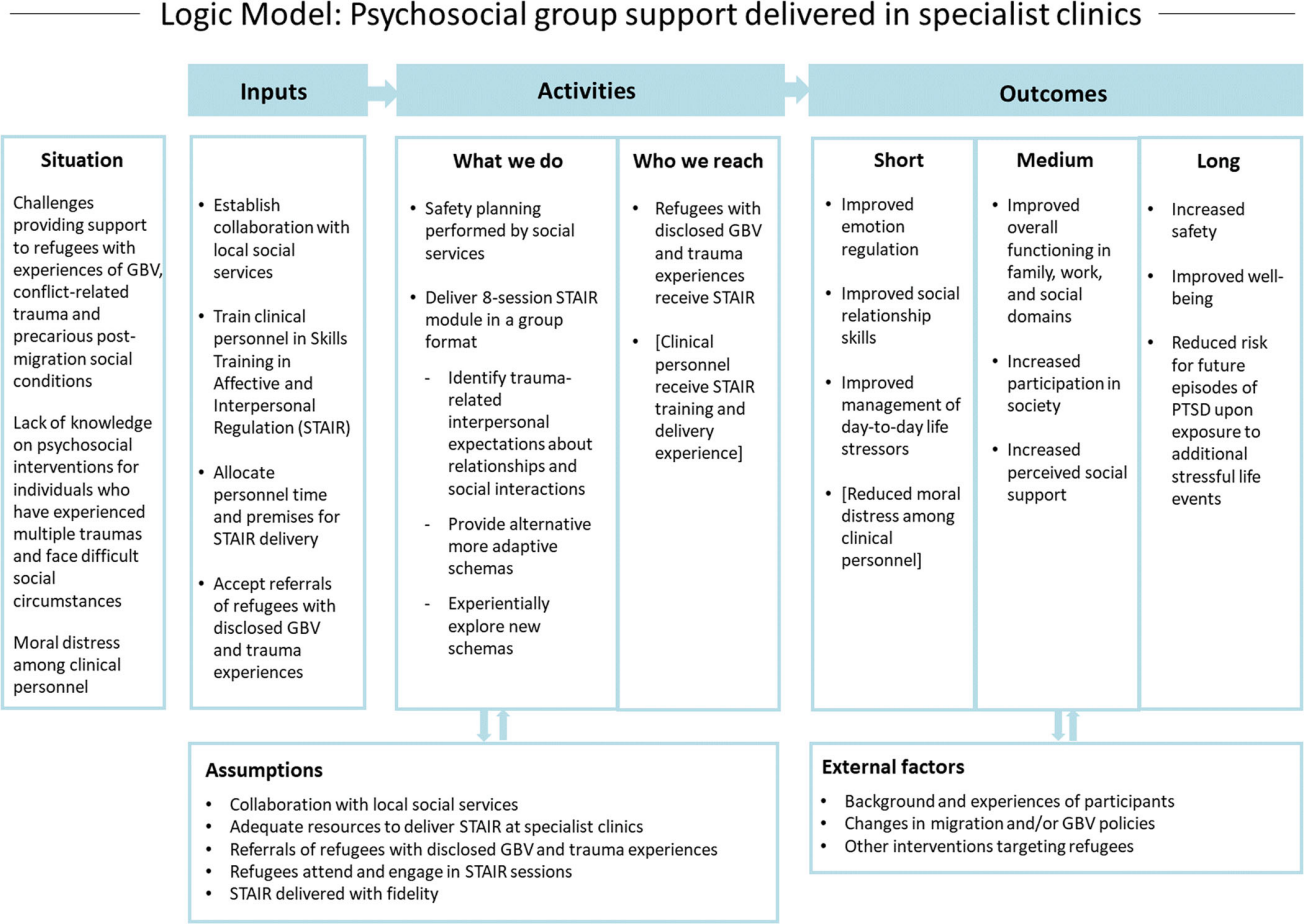
Logic model of psychosocial group support delivered in specialist clinics.

STAIR is not focused on processing trauma but trains the participants in emotion regulation, social relationship skills and skills to manage everyday life and focus on the present [63]. This was regarded as beneficial given the aforementioned challenges clinicians in our team perceived in delivering trauma treatment to refugee victim‐survivors preoccupied by various adverse circumstances. A further motivation for choosing STAIR was that it provides the group‐based format considered important by the survivor co‐researchers without directly targeting trauma in a group setting, which was a concern for the clinicians. The Swedish Red Cross has initiated the introduction of STAIR in one of their treatment centres, which has involved collaboration with local social services and a women's shelter to ensure readiness to respond to the support needs of women with experiences of both migration and violence in near relationships. Collaboration between providers of mental healthcare and social services was regarded as important by our co‐design team and was identified as a promising component in our literature review. Further to this, knowledge exchange with clinicians at NCK will support the implementation, and the co‐design team has raised funds to evaluate the STAIR pilot. See Figure 4 for a logic model of psychosocial group support delivered in specialist clinics.

## 4 Discussion

This study brought survivor co‐researchers, academic researchers, providers of municipal services and clinicians together in a PPI process aimed at co‐designing a psychosocial support service model for refugees in Sweden who have experienced GBV.

Creating safe spaces for refugee victim‐survivors to acquire information according to their own agendas, from peers and through sensitively conducted service outreach, was a key prioritisation for our co‐design team. The need for interventions that seek to build community connectedness and social support and raise awareness about GBV has been previously pointed out [18, 64]. Thus, a central component of our co‐designed model was the intervention to promote help‐seeking hosted by trusted community sites such as the municipal open preschools. The recommendation to promote peer support and normalisation through group delivery, derived from the experiential knowledge of the survivor co‐researchers and providers, is supported by previous research demonstrating positive experiences of group‐delivered IPV interventions for migrants [54]. The suggestion to adapt intervention material to the target groups' needs and concerns, and use bicultural and bilingual staff, aligns with a review on IPV reduction and prevention interventions for pregnant ethnic minority and immigrant women that suggested such strategies could improve intervention reach and participant engagement [53]. In collaboration with survivor co‐researchers and relevant services such as municipal open preschool, the Swedish Red Cross and NCK, further funding has been raised to refine and pilot test the help‐seeking intervention. This will include developing and delivering GBV training for open preschool educators, highlighted in our logic model as a prerequisite for holding the intervention.

However, a meta‐synthesis on refugee women's experiences and coping mechanisms for GBV has pointed out that efforts to provide refugees with opportunities to acquire information from and connect with services will not be sufficient to prevent violence and promote access to support; it is also necessary to address underpinning structural violence, racism and policies [18]. Previous international research has shown that racism can shape experiences of and access to support for GBV [11]. A literature review on racism in Swedish service provision found studies demonstrating experiences of suspicion and neglect, stereotyping, and harassment, and concluded that racism operates through public institutions in Sweden at different areas and societal levels including healthcare, schools and social services [65]. Further research examining how racism influences ideas around GBV and gender, and how this affects victim‐survivors’ access to support would be valuable. Diversity, equity and inclusion training, encompassing antiracism training, should be considered for authorities and service providers in different sectors, including migration services as well as education and healthcare. Further, a study from Sweden, Britain and Denmark found that the dependency and lack of autonomy caused by asylum policies and practices—such as spousal visas, poverty or remote housing—increased vulnerability to GBV and compounded the negative psychological and emotional consequences of GBV [66]. This highlights the need to critically examine existing policies and consider changes to decrease the risk of upholding GBV. The same article reported that psychologists working with people who had experienced persecution and torture felt they could not effectively provide support because their patients were too affected by their migration‐related precarious social conditions to be able to engage [66]. Similarly, research has found perceptions among Swedish social workers that practical support needs might interfere with the provision of treatment to women exposed to IPV, particularly women with limited knowledge about Swedish society [32]. These previous studies support our co‐design team's suggestion of increasing collaboration between mental health services and social services—also proposed in a recent qualitative study with Swedish service providers [24]—that will be further explored in the pilot study of the psychosocial group support delivered at Swedish Red Cross Centres. This need for support to improve living conditions was also the rationale for co‐designing the help‐seeking intervention to include outreach from services such as employment agencies, housing services and legal aid.

When involving co‐researchers from groups who might have experienced marginalisation, stigmatisation and denigration and might have reasons to doubt their own and researchers' capacities to achieve meaningful change—such as refugees with experiences of GBV—it is essential to adopt co‐research practices that strive to uphold reciprocity, self‐determination, transparency and democracy [44]. We found the PiiAF framework helpful in structuring initial discussions aiming to facilitate these important principles and decided to continue using it to guide and document recurrent formative self‐evaluations, and we perceived this application of the framework to work well. Practices pointed out as particularly valuable for meaningful involvement included kindness and recognition of the survivor co‐researchers experiential knowledge, mutual sharing and frequent and inclusive communication, which aligns with what has previously been shown to be important when involving refugees as public contributors [67, 68]. We invested in building the collective understanding necessary for power sharing, allowing the first step of the PPI process to take time and discussing key concepts such as GBV, racism, support and services before moving to prioritising issues and identifying stakeholders to involve. During the formative evaluations, both survivor co‐researchers and researchers reflected on the benefits gained from the partnership. From the survivor co‐researchers' perspectives, the involvement had been an empowering experience, with benefits for themselves as individuals as well as their communities, as they had shared knowledge and insights gained from the project with friends. This aligns with previous literature arguing that co‐production, when carried out with care, can promote healing and agency [44, 61]. From a researcher's perspective, working in partnership with public contributors was not only valuable in terms of the important insights but it was also a source of energy helpful for carrying out the research.

A PPI process such as ours requires time, resources and commitment. We were able to allocate some funding within the research group, generated through teaching activity, as seed money for the first step of our process, thus enabling involvement to start at the conceptualisation stage of the research. Later, our steering group received a research planning grant to fund the PPI workshops with service providers. The various outcomes of our process illustrate the value that can be gained from such efforts to bring public contributors (from seldom‐heard groups) and different stakeholders together in research planning and co‐design. Providers expressed that this PPI process had been a positive example of how users and providers can interact constructively when necessary preparations have contributed to building long‐term cooperation and ensuring a clear definition of common goals. Discussing with public contributors has led to many valuable insights on their services. One example is the adaptation of the information on violence in near relationships provided by the governmental webpage for refugees and asylum‐seekers, a process directly initiated by the service providers in response to the insights gained from this research. Drawing on the collaborations established in this project, NCK and survivor co‐researchers provided feedback on the revised information. The insights and collaborations stemming from this PPI process also led to the initiation of several research projects. These include a survey on CO course leaders' readiness to respond to GBV and an interview study exploring the training needs of open preschool personnel.

The survivor co‐researchers (H.A., I.A., M.G. and Z.A.M.) were essential in designing and carrying out this PPI process, in developing constructive ideas for improving service access, and in disseminating outputs through their own initiatives and channels as well as through participating in researcher‐initiated dissemination. Their contributions add to previous examples of the feasibility and value of involving migrant populations in research and service development [69] and have inspired public involvement in other research and services. Namely, the Swedish Red Cross is using their positive experiences from this project to inform the design of a Swedish Red Cross research project concerning interventions for refugee women, to prepare the implementation of peer supporters at Swedish Red Cross treatment centres, and to establish a platform for user involvement in research at the Swedish Red Cross Competence Centre. Our PPI process exemplifies the theory that efforts to build trust and share decision‐making in research bringing different stakeholders together can help establish sustainable partnerships, which in turn can lead to collaborative health improvement efforts, spin‐off projects and systematic transformation [70].

## 5 Conclusions

Improving psychosocial support for refugees in Sweden affected by GBV requires safe spaces to connect with peers and familiarise them with available services, laws and rights in the society. Further, strengthened collaborations across sectors are necessary to meet the variety of needs. Refugee victim‐survivors are amply capable of identifying areas for service improvement and have valuable contributions to make to health and service research. Investing time and resources in bringing people with lived experiences and different service providers together in co‐design workshops can lead to benefits for those involved and be an effective way to initiate research projects and changes in practice. Recurrent formative self‐evaluations reflecting on working methods, motivations for involvement and support needs can be helpful for involvement processes and serve to sustain relationships and ethical co‐research practices.

## Author Contributions

**Anna Pérez‐Aronsson:** conceptualisation (lead), investigation (lead), writing–original draft (lead), writing–review and editing (lead). **Elin Inge:** investigation (supporting), writing–review and editing (supporting). **Heba Alanbari:** conceptualisation (supporting), investigation (lead), writing–original draft (supporting). **Iman Alsalamah:** conceptualisation (supporting), investigation (lead), writing–original draft (supporting). **Miras Ghannoum:** conceptualisation (supporting), investigation (lead), writing–original draft (supporting). **Zozan Abu Mohammad:** conceptualisation (supporting), investigation (lead), writing–original draft (supporting). **Frida Johansson Metso:** investigation (supporting), writing–original draft (supporting). **Frida Holmqvist:** investigation (supporting), writing–original draft (supporting). **Johanna Belachew:** investigation (supporting), writing–original draft (supporting). **Tove Filén:** investigation (supporting), writing–review and editing (supporting). **Frida Pålsson Hennoks:** investigation (supporting), writing–review and editing (supporting). **Anna Sarkadi:** conceptualisation (supporting), funding acquisition (supporting), supervision (supporting), investigation (supporting), writing–review and editing (supporting). **Georgina Warner:** conceptualisation (lead), funding acquisition (lead), supervision (lead), writing–original draft (supporting), writing–review and editing (lead).

## Conflicts of Interest

The authors declare no conflicts of interest.

## Data Availability

With the safety and comfort of everyone involved in this process in interest, we have agreed that we will not make our notes and material from meetings and workshops publicly available. Any requests regarding material should be directed to the corresponding author.
